# Integrative Profiling for BBB Permeability Using Capillary Electrochromatography, Experimental Physicochemical Parameters, and Ensemble Machine Learning

**DOI:** 10.3390/ijms27010328

**Published:** 2025-12-28

**Authors:** Justyna Godyń, Jakub Jończyk, Anna Więckowska, Marek Bajda

**Affiliations:** 1 Department of Physicochemical Drug Analysis, Faculty of Pharmacy, Jagiellonian University Medical College, 9 Medyczna Street, 30-688 Krakow, Poland; justyna.godyn@uj.edu.pl (J.G.); anna.wieckowska@uj.edu.pl (A.W.); 2 Department of Medicinal Chemistry, Faculty of Pharmacy, Jagiellonian University Medical College, 9 Medyczna Street, 30-688 Krakow, Poland; jakub.jonczyk@uj.edu.pl

**Keywords:** blood–brain barrier, permeability, drug development, in vitro methods, capillary electrochromatography, physicochemical parameters, machine learning

## Abstract

Profiling the blood–brain barrier (BBB) permeability of bioactive molecules during early drug development is critical for optimizing their pharmacokinetic profile. The in vivo ability of a compound to cross the BBB is measured by the log BB parameter; however, its determination requires costly and time-consuming animal experiments. This study aimed to develop a novel in vitro method for high-throughput prediction of log BB values. The approach combines experimental data from open-tubular capillary electrochromatography (CEC) and automated potentiometric titrations, including the CEC retention factor (*k*′), electropherograms, and physicochemical parameters pK_a_ and log D_7.4_. The *k*′ parameter reflects BBB permeability using a capillary internally coated with liposomes that mimic a biological membrane. Preliminary CEC analyses were conducted for 25 neutral drugs at pH 7.4, revealing a promising correlation between the permeability parameters log *k* and log BB. The validation was extended to 57 ionized drugs, with additional determination of pK_a_ and log D_7.4_. A regression model was developed: log BB = −2.45 + 0.1*k*′ *+* 0.3logD_7.4_ + 0.27pK_a_ (R^2^ = 0.64). Furthermore, the analysis of CEC electropherograms enabled the machine learning-based rapid classification of compounds using Dynamic Time Warping, k-Nearest Neighbors, and the Bag-of-SFA-Symbols in Vector Space model, yielding an accuracy of 0.81 and an F1_weighted_ score of 0.8.

## 1. Introduction

Optimization of physicochemical properties and hence the pharmacokinetic profile is a critical step in the drug discovery process, as favorable ADME properties (absorption, distribution, metabolism, and excretion) determine the in vivo fate of a molecule. A key element of pharmacokinetic assessment involves evaluation of the compound’s permeability across biological membranes, particularly the blood–brain barrier (BBB), which is of critical importance. This applies both to compounds intended to act within the central nervous system (CNS) and to peripheral agents, due to their potential for CNS-related side effects.

The BBB is a highly selective structure that separates the brain from systemic circulation. It is formed by the walls of brain capillaries and is characterized by specialized tight junctions between endothelial cells of the brain vasculature. The highly protective properties of the BBB are the result of a complex interaction of a physical barrier (with tight junction restricted paracellular diffusion), transport barrier (via carrier-mediated flux mechanisms), and metabolic barrier (because of enzymes that modify or degrade compounds during transit) [1,2]. As a result, the BBB presents a significant challenge for xenobiotics and serves as a crucial issue in the development of novel therapies.

**Figure 1:**
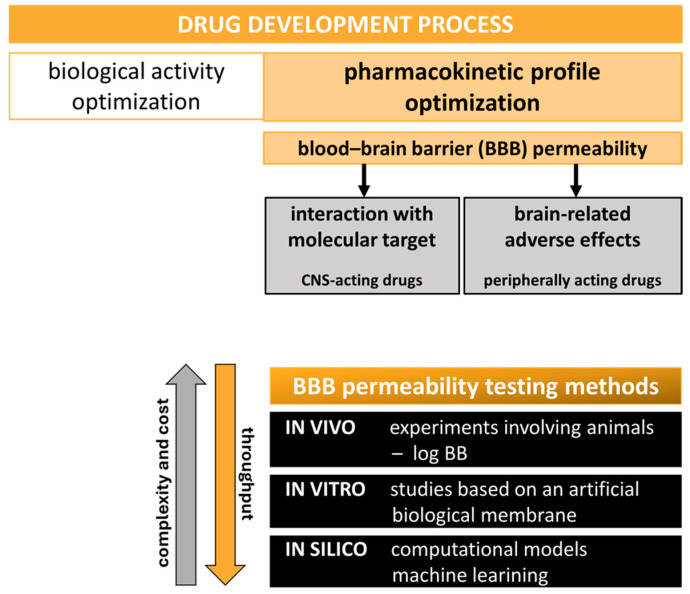
BBB permeability testing as a key step in the drug discovery pipeline. Methods used in the assessment of brain penetration.

While direct in vivo measurement of a compound’s brain penetration remains the most accurate and reliable approach, it requires animal sacrifice and is often labor intensive, expensive, and time consuming [3,4]. Therefore, at the early stages of drug development, high-throughput methods are widely employed to investigate BBB penetration and reduce the need for animal-based experiments. The brain-to-blood concentration ratio at a steady state, represented by the log BB parameter, is a popular endpoint for large-scale BBB permeability predicting models [5,6,7]. Log BB reflects a compound’s actual ability to penetrate the CNS. It is derived from in vivo experiments and typically involves complex studies on rats, requiring also the expertise of skilled scientific staff [8]. In recent years, many approaches have been proposed to be used at the early hit-to-lead drug discovery, including in vitro and in silico methods, as well as their combinations [8,9,10,11]. These simplified methods are sufficient for effective screening of large compound libraries within a short timeframe (Figure 1). Among the in vitro approaches, many tend to assess only the passive diffusion of compounds from the bloodstream into the brain, as this is the most prevalent route for the passage of small molecules into the CNS [12]. Currently, one of the most widely used in vitro assays for estimating passive permeability into the CNS is the parallel artificial membrane permeability assay for the BBB (PAMPA-BBB) [13,14]. Despite its advantages, the PAMPA permeability evaluation is based on the simplified monolayer surrogate membrane prepared from lipids dissolved in dodecane, making the assay conditions significantly different from physiological ones. Next to the PAMPA-BBB, chromatographic-separation-based techniques are of great interest, utilizing immobilized artificial membrane chromatography or electrophoretic approaches [15,16]. Capillary electrophoresis (CE)-based methods are likely to mimic natural conditions. The CE variants (e.g., micellar electrokinetic chromatography, MEKC; liposome electrokinetic chromatography, LEKC; and capillary electrochromatography, CEC) utilize particles like micelles or liposomes as good structural analogues of a biological membrane [17,18,19]. Apart from the bilayer nature of the created surrogate membrane, the CE methods are simple and fast, present high separation efficiency, require only milliliters of solvents, and utilize environmentally friendly aqueous background electrolytes, making them promising PAMPA-BBB alternatives.

The brain penetration of a drug candidate is dependent on the compound’s physicochemical features, such as lipophilicity, ionization state, topological polar surface area, molecular weight and size, conformational flexibility, and hydrogen-bonding capacity. The correlations between permeability and physicochemical properties have been extensively tested based on the large databases of BBB-permeable marketed drugs and research compounds [20,21]. In today’s drug discovery research, many models used to estimate the permeability of drug candidates across the BBB are created based on molecules’ physicochemical characteristics, like pK_a_, log P, log D, and others, to generate predictive equations enabling high-throughput permeability screening as a starting point for prioritizing CNS-active candidates [22,23]. Lipophilicity was among the first molecular descriptors found to play a major role in determining CNS drug availability. For neutral or non-ionized molecules, it is expressed by the octanol–water partition coefficient (log P). For ionizable compounds, lipophilicity varies with pH and is reflected by the pH-dependent octanol–water distribution coefficient (log D). Over the years many log P and log D ranges have been proposed for drug candidates as optimal for brain exposure [21], starting with the Rule for CNS drugs (RoCNS) requirements proposed in 1999 [24] as the variant of Lipinski’s Rule of Five (Ro5)—a widely accepted threshold for orally bioavailable candidates but not strict enough for CNS agents [20]. Next to the lipophilic properties, the acid–base profile of the molecule may strongly affect the BBB permeability. A compound’s acidity, described by the pK_a_ value (negative logarithm of acid dissociation constant), determines a molecule’s ionization state at a given pH, which in turn affects its ADME properties, as only unbound and unionized species may freely passively diffuse across membranes [25]. Usually, the in silico calculated log P/log D and pK_a_ values are utilized in permeability-predicting algorithms, as they are easy to obtain for large libraries of compounds [26,27,28]. However, in vitro experimentally determined physicochemical parameters are often more valuable, as they reflect the true molecular characteristics, providing a more reliable input for predictive models and drug development decisions [29,30,31]. The traditional experimental methods used to determine physicochemical properties are, however, time consuming and labor intensive and may require a high consumption of solvents and co-solvents, as well as large samples of test compounds. These are potentiometric manual titrations, as well as spectrophotometric and conductometric approaches used for pK_a_ determination and a widely accepted shake-flask method for determining the compound’s lipophilicity [32,33]. Over the years, many experimental high-throughput methods have been proposed for physicochemical property determination based on the separation techniques as well as spectrometric and titration approaches [31,34]. In early 2000, the Sirius instrument was developed, serving fast and automated experimental potentiometric titrations for pK_a_ and log P determination [31,34,35]. This approach requires only milliliters of solvents and 1–2 mg of research compound. Moreover, poorly soluble compounds may be easily tested in a water–cosolvent environment. The apparatus works in a wide pH range (pH 2–12), and even overlapping pK_a_ values can be reliably determined [31].

Next to the in vitro experimental methods, the in silico computational approaches are useful in BBB permeability assessment at the early drug discovery stage. In recent years, various computational machine learning (ML) methods have been employed to estimate brain penetration. Such approaches offer an inexpensive and time-efficient strategy for evaluating the CNS permeability of novel lead compounds [36,37,38]. ML-supported prediction of brain exposure relies on the use of computational algorithms to develop predictive models that, based on molecular and biological data, can estimate the likelihood of a compound crossing the BBB. These models are trained on datasets of compounds with known BBB permeability profiles, enabling the identification of complex relationships between molecular descriptors and BBB penetration potential [39,40]. Among the most widely used ML methods are Support Vector Machine, Random Forest, k-Nearest Neighbors (k-NN), Multidimensional Linear Regression, Linear Discriminant Analysis, and the more specialized Bag-of-SFA-Symbols in Vector Space (BOSSVS) algorithm, suited for time series classification [36,41].

In the group of ML strategies, ensemble learning techniques have shown promise in BBB permeability prediction. By combining the outputs of multiple base learners, ensemble models reduce variance and bias, often outperforming individual classifiers in terms of accuracy, robustness, and generalization to unseen data [37,42,43]. Their ability to capture diverse patterns in high-dimensional molecular data makes them especially suitable for modeling the complex mechanisms governing BBB transport.

In our previous studies, preliminary research was undertaken to develop a CE-based, novel, alternative method for estimating passive brain permeability. In the first work, the MEKC—a variant of CE—was applied to estimate the lipophilicity of bioactive compounds [44]. Next, for BBB permeability studies, we proposed the use of open-tubular capillary electrochromatography (open-tubular CEC), which, to the best of our knowledge, has never been applied for this purpose. Open-tubular CEC combines conventional CE separation in an electric field with a capillary internally coated with a stationary phase typical of chromatographic systems. The inner coating consists of liposomes with a phospholipid bilayer structure, effectively mimicking natural biological membranes. Apart from the surrogate membrane structure, the CEC technique offers more advantages over the PAMPA-BBB assay. The separation conditions, including the absence of organic solvents, more closely resemble the physiological environment. The method is straightforward to perform and precise and requires only ~1.5 mg of analyte (not necessarily pure, unlike in PAMPA) and a small aliquot of reagents. Using CEC, we conducted preliminary permeability studies on 25 neutral or non-ionized drugs at pH 7.4, obtaining promising results [45]. We demonstrated a comparable correlation between permeability parameters obtained via CEC (log *k*) and those from PAMPA-BBB (log P_e_), with the experimental log BB parameter reflecting a compound’s in vivo ability to penetrate the CNS. Using log *k* values from CEC, we classified the 25 compounds as either low (CNS(−)) or high (CNS(+)) brain-penetrating. The classification matched literature-based CNS permeability data in 75% of cases, compared to 77% agreement using PAMPA-BBB.

The aim of the presented study was to complete the validation of the CEC-based method. This involved electrophoretic analysis of an additional set of 57 compounds ionized at pH 7.4. Permeation-related *k*′ values were calculated from separations using both liposome-coated and uncoated capillaries. To achieve a satisfactory correlation between *k*′ and log BB parameters for the entire dataset of 57 ionized references, additional descriptors were included in the analysis. As the membrane permeability is highly dependent on compounds’ physicochemical properties, the pK_a_ and log D_7.4_ values were experimentally determined with automated pH-metric titrations and then used to evaluate a simple linear regression. The obtained correlation allows the estimation of log BB values from in vitro parameters. Furthermore, we used CEC electropherograms from initial [45] and validation experiments to create an ML-based model that enables the rapid qualitative classification of tested compounds according to their ability to penetrate the BBB. By utilizing a combination of Dynamic Time Warping (DTW) with the k-NN algorithm and BOSSVS model, an ensemble method was developed to achieve exceptional classification performance. Our findings resulted in the development of a new analytical tool to support the discovery and optimization of novel drug candidates.

## 2. Results and Discussion

### 2.1. CEC-Derived Permeability (k′) and Automated Sirius T3 Physicochemical Profiling (pK_a_, Log P, and Log D_7.4_)

In the presented study, we used 57 commercial reference drugs, with diverse reported BBB permeability (experimental in vivo log BB values taken from [46,47,48,49]) and diverse acidity and lipophilicity but all ionized in CEC analysis conditions (pH 7.4). For all tested drugs, we determined in vitro permeability *k*′ coefficients and physicochemical properties defined by pK_a_, log P, and log D_7.4_ values. All experimental results are collected in Table 1 along with experimental log BB in vivo data.

In CEC experiments, the migration times of drugs depend on two phenomena: the electroosmotic flow (always with the direction towards the negatively charged cathode [50]) and the electrophoretic migration of the analyte in the applied electric field, comprising its interaction with the inner liposomal layer. The analyzed drugs should interact with the coating mimicking a natural membrane by entering the liposomes with speed and exposure depending on the compounds’ physicochemical parameters and hence their membrane permeation ability. However, the migration parameters of the ionized references were mainly driven by the electric field, rendering interactions with liposomes negligible and thus hindering differentiation among the tested compounds. Therefore, the migration times obtained using the liposome-coated capillary needed to be complemented with those determined in the uncoated variant, and both results were included in the *k*′ permeability parameter calculations.

The acidity and lipophilicity properties for 57 reference drugs were evaluated by the experimental determination of pK_a_ and log P values, respectively, using potentiometric titrations. Log D_7.4_ values were calculated based on the experimental pK_a_ and log P data.

### 2.2. Quantitative Log BB Estimation—Correlation of In Vitro k′, pK_a_, and Log D_7.4_ with In Vivo Data

Our preliminary research [45] yielded promising results, enabling the establishment of a correlation between the compound’s permeability parameter (log *k*) determined by CEC and the corresponding in vivo log BB value, with R^2^ = 0.43 for a set of 25 reference drugs, neutral or unionized at pH of 7.4.

Further validation studies, described in this paper, were conducted with an additional set of 57 compounds, ionized at a physiological pH. For these ionized drugs, establishing a satisfactory correlation between in vitro and in vivo permeability data required the inclusion of additional parameters in the linear regression calculations. This adjustment was necessary due to the pronounced influence of molecular charge on CEC migration behavior and, consequently, on *k*′ values, when analyzed under the applied voltage. To account for the interactions of the tested reference compounds with the liposomal surrogate membrane employed in CEC, *k*′ values were determined using both coated and uncoated capillary variants. In addition to the experimentally derived *k*′ values, the key physicochemical parameters pK_a_, log P, and log D_7.4_ were experimentally determined. The obtained in vitro data were used in the Ordinary Least Squares (OLS) regression analysis (statsmodels [51]). The final correlation is described by Equation (1), with R^2^ = 0.64, F = 31.32, and *p* = 8.7 × 10^−12^,

(1) $$logBB=-2.45+0.1\times k^{\prime }+0.3\times logD_{7.4}+0.27\times pK_{a}$$

where log BB is the in vivo permeability parameter calculated as the logarithm of the brain-to-plasma concentration ratio and *k*′ is a CEC permeability parameter calculated based on Equation (2) (see Section 3.4.1).

**Figure 2:**
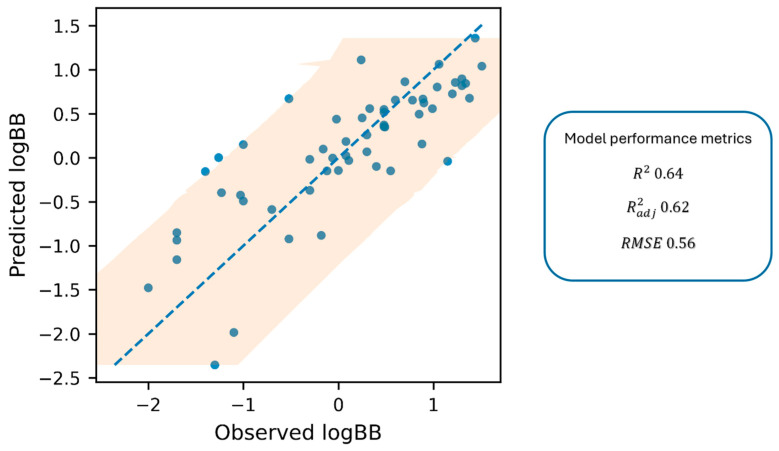
The parity plot displays predicted log BB against observed log BB values, including a 1:1 dashed blue line and a 95% prediction interval (shaded area).

Figure 2 shows the parity plot (predicted vs. observed values), with the 1:1 line and 95% prediction interval.

Based on the established correlation, the log BB parameter, reflecting the actual ability of a compound to penetrate the brain, can be reliably estimated from in vitro experimental data. This correlation provides a rapid and practical tool for predicting membrane permeability, a key determinant of ADME behavior. The proposed model offers valuable support at the early stage of drug discovery, enabling efficient screening of large compound libraries.

While the proposed Equation (1) explains a considerable proportion of the log BB variance (R^2^ = 0.64), the remaining unexplained variability is not unexpected for BBB endpoints and can be attributed to both methodological and biological factors. Because the experimental log BB values were compiled from the prior literature, they inevitably aggregate inter-study differences (e.g., protocol-, timing-, and reporting-dependent effects), which introduces label noise that cannot be eliminated by regression on in vitro descriptors alone. Moreover, it should be emphasized that log BB is a multifactorial parameter, reflecting not only passive permeability but also contributions from efflux and influx transporters, brain metabolism, and tissue binding [3]. The current predictor set (*k*′, pK_a_, and log D_7.4_) predominantly captures passive membrane interaction/partitioning; therefore, deviations are expected for compounds whose BBB disposition is influenced by mechanisms not encoded by these variables. To mitigate this limitation, the set of 57 compounds was deliberately selected to minimize the contribution of active transport at the BBB and to reflect CNS-relevant chemical space (45 positively charged and 12 negatively charged references). The vast majority of these compounds have not been reported as strong substrates of major efflux transporters (e.g., P-glycoprotein) or active influx transporters, supporting the interpretation that the predicted log BB values primarily reflect passive permeability. Consequently, the developed model is best suited for compounds with negligible transporter affinity, and preliminary screening for efflux or influx using Caco-2, MDCK-MDR1 [52] or similar assays is recommended when transporter involvement is suspected. In this context, the few compounds exhibiting larger residuals in the parity plot should be interpreted as expected outliers arising from endpoint heterogeneity or residual non-passive determinants rather than as failures of the experimental workflow.

### 2.3. Qualitative Log BB Estimation—Machine Learning-Based Model

**Figure 3:**
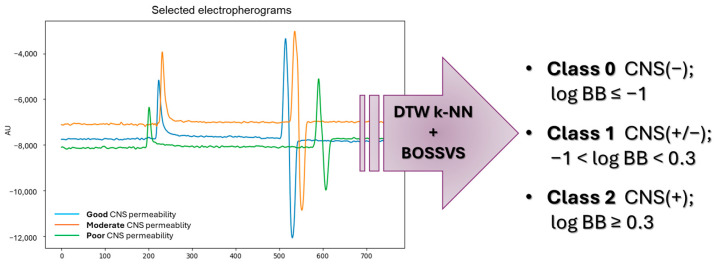
Qualitative prediction of brain permeability—estimation of log BB based on the CEC electropherograms by ensemble learning algorithm combination.

We next assessed whether electropherogram shape alone could provide a fast, qualitative indication of BBB permeability. Electropherograms obtained from the present experiments and previous studies [45] were used as fixed-length time series (Figure 3). For this purpose, we applied the ensemble learning hybrid approach, combining two complementary time-series classifiers: a shape-based k-NN model, employing DTW as the distance function, and a motif-based BOSSVS algorithm.

**Figure 4:**
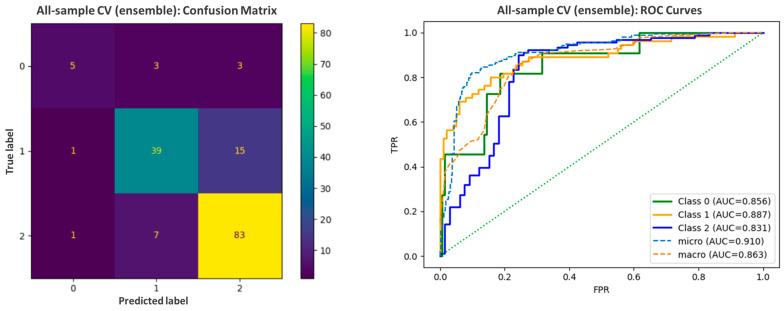
The error matrix and ROC curves prepared for the assessment of the predictive abilities of the ML-based model.

Despite the limited dataset, the developed classifier demonstrated a good accuracy between 0.75 and 0.87 per fold and F1_weighted_ in the range of 0.74 to 0.87 during 5-fold cross-validation. When summarized as a single estimation over all samples, accuracy was 0.81, with balanced accuracy reaching 0.69, and F1_weighted_ was equal to 0.8. For evaluation purposes, the error matrix and ROC curve analysis were prepared (Figure 4). For 11 CNS non-permeable references (Class 0), 45.46% were correctly classified as BBB (−) and another 3 (27.27%) as BBB (+/−). For 91 CNS highly permeable drugs forming the largest class (Class 2), 83 (i.e., 91.21%) were predicted correctly as BBB (+), 7 as BBB (+/−), and only 1 incorrectly as a nonpermeable drug. In the case of Class 1 (moderate permeability), nearly 70.91% references (39 out of 55) were correctly assessed, with 15 classified as BBB (+), and again only 1 was mistaken as nonpermeable.

The optimized ensemble consistently favored BOSSVS (final weight for k-NN was 0.05), suggesting that symbolic motif content extracted by SFA-based words is the dominant signal in collected electropherograms. BOSSVS captures recurrent local shapes and relative intensities that could reflect compound-dependent features (charge state, electrophoretic mobility shifts, and matrix effects), while being robust to small time shifts. Nevertheless, the addition of the DTW k-NN, which focuses on global shape alignment, resulted in a significant increase in performance metrics, including an increase in accuracy from 0.66 to 0.81 and an F1_weighted_ score from 0.62 to 0.80.

## 3. Materials and Methods

### 3.1. Chemicals

References (purity > 99.5%) were purchased as follows: acetylsalicylic acid, albuterol sulfate, amitriptyline hydrochloride, atenolol, atropine sulfate, betahistine dihydrochloride, bromperidol, buspirone hydrochloride, chlorambucil, chlorpromazine hydrochloride, citalopram hydrochloride, clonidine hydrochloride, clozapine, desipramine hydrochloride, diclofenac sodium, donepezil hydrochloride, fluphenazine dihydrochloride, galanthamine hydrobromide, haloperidol, hydroxyzine dihydrochloride, ibuprofen, imipramine, indomethacin, ketorolac tris, loperamide, levofloxacin, metoclopramide hydrochloride, metoprolol tartrate, mepyramine maleate, mianserin hydrochloride, naproxen, nicotine hydrogen tartrate, nortriptyline hydrochloride, paroxetine hydrochloride, phenylbutazone, physostigmine salicylate, pindolol, promazine hydrochloride, propranolol hydrochloride, ranitidine hydrochloride, risperidone, rivastigmine tartrate, ropinirole hydrochloride, thioperamide maleate, thioridazine hydrochloride, trazodone hydrochloride, trifluoperazine dihydrochloride, triprolidine hydrochloride, quinidine, venlafaxine hydrochloride, verapamil hydrochloride, zidovudine, and zolmitriptan (Sigma Aldrich, Steinheim, Germany); codeine phosphate (Fagron, Kraków, Poland); cyclobarbital calcium and phenobarbital sodium (Polfa Tarchomin, Warszawa, Poland); and salicylic acid (Galfarm, Kraków, Poland).

1-Octanol (HPLC-grade), 2-propanol, hydrochloric acid (HCl) 0.5 M, potassium hydroxide (KOH) ampule 0.5 mol/L, methanol (hypergrade for LC-MS), chloroform (HPLC purity), sodium hydroxide (NaOH, 1.0 M, CE purity), Triton X-100, dimethyl sulfoxide (DMSO, HPLC-grade), HEPES buffer (40 mM, pH adjusted to 7.4 by addition of 1.0 M NaOH), pH 7 buffer (Mettler Toledo, Columbus, OH, USA), potassium chloride, potassium hydrogen phthalate, dipotassium hydrogen orthophosphate, 1-palmitoyl-2-oleoyl-*sn*-glycero-3-phosphocholine (POPC), and 1,2-diacyl-*sn*-glycero-3-phospho-L-serine (PS) were obtained from Sigma Aldrich (Steinheim, Germany). T3 Neutral Linear Buffer was provided by Pion (Forest Row, UK). Hydrochloric acid (HCl) 36% (*v/v*) and 0.5 mol/L was purchased from Stanlab (Lublin, Poland) and Sigma Aldrich (Steinheim, Germany), respectively. Purified water was derived from the deionization unit (Hydrolab, Straszyn, Poland). Nitrogen (4.8 technical grade, 99.998%) was purchased from AirProducts (Warszawa, Poland). HEPES buffer and HCl were filtered (0.45 μm pore size filter) before use.

### 3.2. Materials

Polycarbonate filter units of 0.1 μm pore size and syringe filter units with a pore size of 0.45 μm were purchased from Sigma Aldrich (Steinheim, Germany). Uncoated fused-silica capillary (Beckman Instruments, Fullerton, CA, USA) with a 59.1 cm × 50 µm internal diameter and a 375 µm external diameter with an effective length of 49 cm was used throughout the study.

For automated titrations, T3 Pion vials (Pion, Forest Row, UK) were utilized.

### 3.3. Instruments

All CEC analyses were performed on a P/ACE MDQ instrument with a DAD detector (Beckman Instruments, Fullerton, CA, USA). The UV detection of the analytes was carried out at a wavelength of 220 nm. We used 32 Karat Software version 8.0 to record and analyze the obtained electropherograms. Liposome extrusion was performed using LiposoFast Liposome Factory (Avestin Europe, Mannheim, Germany).

The Sirius T3 device from Pion (Forest Row, UK) was used to determine the physicochemical properties of the tested references by automated pH-metric titrations.

Computations were performed on a Linux workstation running Ubuntu 22.04 LTS equipped with a 12-core AMD Ryzen Threadripper 1920x CPU, an NVIDIA GeForce GTX 1070 GPU, 32 GB RAM, and 64 GB of dedicated swap space.

Publicly available Python (v3.12) libraries—pandas (v2.0.3), scikit-learn (v1.2.2), matplotlib (v3.7.1), openpyxl (v3.1.5), sktime (v0.39.0), and pyts (v0.13.0)—were used for data cleaning, model training and testing, and subsequent evaluation and interpretation.

### 3.4. Methods

#### 3.4.1. CEC—Determination of Permeability k′ Parameter

The CEC method optimized in our preliminary studies [45] was used in the current research. The separation HEPES buffer, POPC/PS liposomes (80:20 mol% ratio; large unilamellar vesicles type; LUV), and test samples were prepared according to the protocol described earlier [45]. The capillary coating procedure was maintained as previously optimized, involving rinsing the capillary for 10 min with 0.5 M HCl under a pressure of 93.8 kPa followed by 15 min with water and finally 10 min with the 3 mM liposome solution, all under a pressure of 93.8 kPa. To complete the coating procedure, the capillary was left to stand filled with the liposome solution for 15 min.

To assess the efficiency of the capillary coating procedure, the electroosmotic flow (EOF) in the coated and uncoated capillary variants was compared. Electroosmotic mobility (μ_EOF_) was determined using methanol as a neutral marker due to its minimal interaction with the capillary wall. In the coated capillary, a significant suppression of EOF was observed, as evidenced by a prolonged methanol migration time (t_EOF_) and hence a reduced μ_EOF_ (Table 2).

The obtained liposomal POPC/PS layer mimicked the natural biological membrane due to the bilayer nature of the LUVs’ structure and obtained viscoelastic SVL (supported vesicular layer) type of coating, as confirmed by earlier studies [53,54,55] and described also in our preliminary research [45]. As the coating well reflects the structure of the natural membrane, the *k*′ parameter obtained from the CEC analyses represents the permeability coefficient of compounds through the blood–brain barrier.

Since the reference drugs tested in this study are compounds ionized at pH 7.4, their migration times determined in the uncoated and coated capillary variants were used in the *k*′ permeability parameter calculations. At first, the experiments were conducted in the liposome-untreated capillary. Next, after coating the capillary with the inner liposomal layer, the migration times of analytes were evaluated once again in the coated one. Experiments in both uncoated and coated variants of the capillary were conducted at room temperature using the normal polarity mode under 20 kV of voltage and 10 μA of current, keeping the parameters optimized in preliminary research studies [45] and resulting in approx. 3–7 min and 3–11 min of migration times, respectively, depending on the capillary variant, always shorter in the untreated one. The pH 7.4 HEPES buffer (40 mM) was used as the background electrolyte solution in all CEC experiments.

The CEC permeability *k*′ parameter was calculated for each reference drug based on its experimentally determined migration times in the uncoated and coated capillary variants using Equation (2) [56]:

(2) $$k^{\prime }=t_{R}(1/t_{EOF}+\;1/t_{R^{\prime }}-\;1/t_{{EOF}^{\prime }})-1$$

where t_R_ and t_EOF_ are the migration times of the analyte and EOF marker in the coated capillary, respectively, and t_R′_ and t_EOF′_ are analogical migration times in the uncoated capillary.

#### 3.4.2. Automated Sirius T3 Titrations—Determination of Physicochemical Properties (pK_a_, Log P, and Log D_7.4_)

**Figure 5:**
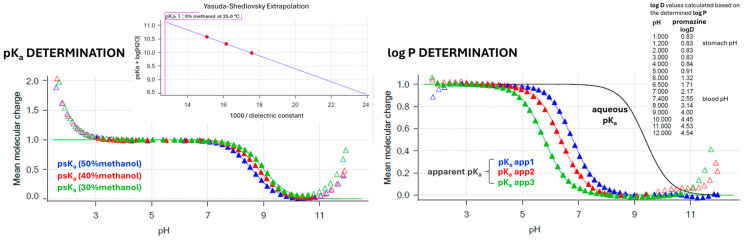
Experimental determination of pK_a_ and log P (log D_7.4_) values by Sirius T3 automated potentiometric titrations. Ionization graphs (Bjerrum curves) are presented for the reference drug promazine as a representative compound.

Using the Sirius T3 device, the physicochemical properties of the reference drugs were determined by automated potentiometric titrations. The compounds’ acidity was measured by determining their pK_a_ parameters. Three independent measurements of pK_a_ values in ionic strength adjusted (ISA) water (0.15 M KCl) were conducted subsequently in one vial. For poorly soluble compounds the cosolvent (methanol) was applied, with three independent measurements of the apparent pK_a_ (p_s_K_a_) conducted with the diverse weight % content of the methanol in assay conditions (water/cosolvent ratios of 70/30, 60/40, and 50/50, respectively). The obtained p_s_Ka values were next extrapolated to the 0% content of methanol (pure ISA water) using the Yasuda–Shedlovsky extrapolation plots. The lipophilicity of the research compounds (log P parameters) was measured by the automated potentiometric method as well, based on the previously obtained compounds’ pK_a_ values. The aqueous pH-metric titrations were carried out in the presence of *n*-octanol—an immiscible water partition solvent. Under these circumstances, a lipophilic sample partitions into the octanol layer and causes a shift to the aqueous phase equilibria, resulting in the appearance of the apparent pK_a_ (p_o_K_a_) (Figure 5). The log P parameters were determined based on the differences between the apparent p_o_K_a_ and aqueous pK_a_ values in three independent titrations conducted with diverse volume ratios of the aqueous and *n*-octanol phases. Log D_7.4_ values were calculated automatically by the Sirius T3 Refine software (version 2.0.0.0) based on the obtained log P and pK_a_ values.

#### 3.4.3. In Silico Ensemble Learning Model

A total of 157 high-quality capillary electropherograms from repeated measurements of 43 drugs were chosen and treated as one-dimensional time series (amplitude vs. migration time). Repeated measurements of the same compound were kept as independent samples to reflect real experimental variability. The labels indicating CNS penetration were categorized based on the log BB value from the literature. The exact cut-off points used were based on earlier publications and applied uniformly to all samples [57]:

- Class 0—poor CNS permeability (log BB ≤ −1);
- Class 1—moderate CNS permeability (−1 < log BB < 0.3);
- Class 2—good CNS permeability (log BB ≥ 0.3).

Labels were encoded to ensure stable class indices throughout training and evaluation.

Raw electropherograms were loaded as fixed-length 1D sequences. Non-numeric entries were coerced to NaN (Not a Number) and removed. Where relevant, the first *N* points of each trace (*N* = 100) were optionally omitted to suppress injection/early-baseline artefacts while retaining the original sample indexing in reports. Each time series was z-normalized per sample to reduce between-run baseline and scale effects without altering within-run morphology.

Model selection and performance estimation followed a nested cross-validation design, with an outer loop using stratified K-fold cross-validation (CV) with the number of folds bounded by the size of the smallest class (up to 10 folds), ensuring each fold contained all classes without over-fragmenting minority classes, and an inner loop for each outer training split with a stratified inner K-fold (up to 3 folds, also capped by the smallest class in the inner training set) used to tune ensemble voting type and weights. The best inner configuration was refitted on the full outer training data and evaluated on the outer validation fold. This provides an unbiased estimate of generalization performance under class imbalance.

Two complementary classifiers based on BOSSVS and k-NN utilizing DTW with the Sakoe–Chiba band as a distance metric [38,58,59] were trained with nested stratified cross-validation to preserve class balance in each fold and static seed 42.

k-NN serves as a shape-based baseline leveraging instance similarity in the original time-series space. In CE data, subtle changes in peak shapes and relative intensities can be informative; k-NN complements the motif-focused BOSSVS by emphasizing global waveform similarity. Unlike traditional metrics that compare data point by point (i.e., Euclidean distance), DTW accounts for temporal distortions and is therefore better suited for analyzing time-dependent data [60].

The k-NN hyperparameters number of neighbors, voting weights, and warping band radius were optimized with grid search during the inner loop. F1_weighted_ score was chosen as the scoring metric to account for class imbalance. The outer loop evaluated model accuracy, balanced accuracy, precision_weighted_, recall_weighted_, and F1_weighted_, alongside confusion matrices and one-vs.-rest ROC curves. For any training set evaluation of k-NN we applied leave-one-out logic, excluding the sample from its own neighbor set to avoid trivial self-matches.

BOSSVS converts a time series into a symbolic representation via Symbolic Fourier Approximation (SFA) and learns a vector space model on the resulting “word” counts. This approach is well suited to electropherograms because it captures local frequency patterns and motifs resilient to minor time warping and noise typical in CE profiles. The BOSSVS hyperparameters window size, word length, and number of bins were optimized with grid search during inner loop tuning with the F1_weighted_ score. The outer loop reports the full metrices accordingly.

To combine the complementary inductive biases of k-NN, DTW, and BOSSVS, we trained the ensemble model, which blends their class-probability outputs ($P_{kNN}$ and $P_{BOSSVS}$) into $P_{ens}$ using a convex weighted average presented in Equation (3),

(3) $$P_{ens}=\omega \;P_{kNN}+\left(1-\omega \right)P_{BOSSVS},\;\omega \;\in [0,1]$$

where $P_{kNN}$ is the k-NN probability output, $P_{BOSSVS}$ is the BOSSVS probability output, $P_{ens}$ is the convex weighted average probability from the ensemble model, and ω is the weight for the k-NN probability output.

To avoid optimistic bias from self-prediction artifacts, base model probabilities were generated out-of-sample by refitting the best k-NN and BOSSVS models only on the fold’s training split using the previously selected hyperparameters. Then, per-fold out-of-fold (OOF) probability matrices for both models aligned to the common label set were produced.

Within the outer-fold training data, an inner CV scanned ω on a coarse grid (e.g., 0.00–1.00 in 0.05 steps) and chose the weight maximizing F1_weighted_. The chosen ω was then applied to blend the outer test probabilities, from which we reported full scoring metrices.

For discrimination analysis, one-vs.-rest ROC curves and AUC were computed. Confusion matrices were produced on the final model for descriptive purposes.

## 4. Conclusions

This study presents a straightforward and efficient method for the early evaluation of blood–brain barrier permeability that integrates experimental techniques, i.e., open-tubular capillary electrochromatography (CEC) and Sirius T3-based automated potentiometric titrations, with machine learning analysis. A robust and reproducible CEC protocol was established using a liposome-coated capillary (POPC/PS composition) to mimic the bilayer structure of biological membranes. The quality of the coating was verified by monitoring the reduction and stability of the electroosmotic flow (EOF). Passive in vitro permeability was measured using the permeability *k*′ parameter, calculated by comparing analyte migration times in coated and uncoated capillaries. Initially validated for 25 neutral drugs, the method was expanded to include 57 ionized compounds at pH 7.4, maintaining the inherent advantages of CE—short analysis time, aqueous buffers, and minimal material consumption.

The results enabled the establishment of a quantitative relationship between in vitro and in vivo permeability. Combining experimentally determined *k*′, pK_a_, and log D_7.4_ parameters yielded a linear model for log BB with R^2^ = 0.64 (F = 31.3; *p* ≈ 8.7 × 10^−12^). This correlation allows rapid, equation-based estimation of in vivo log BB values solely from in vitro data, offering a cost-efficient and ethically favorable alternative to animal testing and a valuable screening tool for early drug discovery. These results expand upon our prior observations with neutral compounds, indicating that the inclusion of ionization and lipophilicity descriptors restores the predictive power of the model for ionized drugs studied under an electric field.

We developed and evaluated a simple probability-level ensemble of two complementary classifiers for CE electropherograms aimed to enable rapid log BB class assignment. DTW-based k-NN captures global shape similarity under elastic time shifts, and the BOSSVS (Bag-of-SFA-Symbols) model encodes local motif structure robust to small temporal drift. Both methods were individually effective. The described workflow aligns with CE’s strengths: minimal sample preparation, rapid runs, and high throughput. Because the models consume the entire trace, analysts are spared peak detection and complex curation—steps that often bottleneck CE pipelines. The BOSSVS-heavy ensemble suggests that motif-level differences in electropherograms—potentially tied to the ionization state (pK_a_), hydrophobicity (log P/log D), and buffer interactions—encode permeability-relevant information that the classifier can exploit. This positions CE + ML as a pragmatic front-end to prioritize compounds for more resource-intensive assays or in vivo studies.

Future extensions of the described methodology, including the expansion of baseline measurements, particularly for poorly permeable compounds, and the exploration of machine learning algorithms incorporating probability calibration, class-specific decision thresholds, and additional time-series learners, are expected to improve minority-class sensitivity and facilitate seamless integration into high-throughput CE workflows.

## Figures and Tables

**Table 1:** Results of experimental analyses. Permeability *k*′ coefficients obtained by CEC method. Physicochemical properties (pK_a_, log P, and log D_7.4_) determined by potentiometric titrations. Log BB parameters collected as published experimental in vivo data [46,47,48,49].

References	CEC	Sirius T3 pH-Metric Titrations			Log BB ^5^
	*k*′ ± SD ^1^	pK_a_ ± SD ^2^	Log P ± SD ^3^	Log D_7.4_^4^	
acetylsalicylic acid	0.03441 ± 0.00987	3.36 ± 0.00	1.31 ± 0.01	−2.73	−1.30
amitriptyline	0.00004 ± 0.00001	9.23 ± 0.08	4.68 ± 0.02	2.84	1.30
atenolol	0.00183 ± 0.00051	9.50 ± 0.01	0.06 ± 0.01	−2.04	−1.00
atropine	0.00440 ± 0.00309	9.84 ± 0.03	1.73 ± 0.01	−0.72	−0.06
betahistine	0.03582 ± 0.00587	9.84 ± 0.00	0.49 ± 0.01	−1.95	−0.30
bromperidol	−0.00795 ± 0.00034	8.45 ± 0.10	3.87 ± 0.05	2.81	1.38
buspirone	0.02096 ± 0.00194	7.62 ± 0.00	2.89 ± 0.02	2.47	0.48
chlorambucil	0.00397 ± 0.00087	4.72 ± 0.04	3.71 ± 0.02	1.07	−1.70
chlorpromazine	0.34688 ± 0.03015	9.07 ± 0.01	5.10 ± 0.03	3.43	1.06
citalopram	0.00405 ± 0.00229	9.43 ± 0.05	3.42 ± 0.00	1.39	0.48
cyclobarbital	0.00186 ± 0.00088	7.71 ± 0.02	1.33 ± 0.01	1.16	−0.30
desipramine	−0.01523 ± 0.00343	10.25 ± 0.02	4.00 ± 0.01	1.35	1.20
diclofenac	0.00802 ± 0.00320	4.19 ± 0.01	4.33 ± 0.01	1.26	−1.70
donepezil	−0.01233 ± 0.00010	9.10 ± 0.02	3.90 ± 0.02	2.19	0.89
fluphenazine	0.11096 ± 0.00192	7.79 ± 0.07	5.12 ± 0.06	4.58	1.51
galanthamine	0.00203 ± 0.00150	8.32 ± 0.00	1.15 ± 0.00	0.18	0.00
haloperidol	−0.00906 ± 0.00138	8.66 ± 0.08	4.37 ± 0.08	3.18	1.34
hydroxyzine	−0.00058 ± 0.00031	7.66 ± 0.04	3.73 ± 0.03	3.34	0.90
ibuprofen	−0.03690 ± 0.00011	4.62 ± 0.05	3.80 ± 0.00	1.07	−0.18
imipramine	−0.04094 ± 0.00432	9.45 ± 0.12	4.44 ± 0.01	2.39	1.30
indomethacin	0.01869 ± 0.00624	4.71 ± 0.01	6.31 ± 0.12	3.92	−1.26
ketorolac	0.02127 ± 0.00131	3.64 ± 0.06	2.90 ± 0.04	−0.05	−2.00
clonidine	0.00321 ± 0.00046	8.12 ± 0.01	1.53 ± 0.01	0.74	0.11
clozapine	0.00693 ± 0.00245	7.58 ± 0.01	3.92 ± 0.10	3.52	0.60
codeine	−0.01188 ± 0.00888	8.22 ± 0.05	1.14 ± 0.01	0.26	0.55
levofloxacin	−0.04345 ± 0.00321	8.13 ^6^ ± 0.00	0.82 ± 0.01	0.77	−0.70
loperamide	0.01470 ± 0.00102	8.69 ± 0.12	4.69 ± 0.17	3.21	0.70
mepyramine	0.00817 ± 0.00067	8.71 ± 0.05	2.73 ± 0.02	1.48	0.49
metoclopramide	−0.00563 ± 0.00136	9.32 ± 0.02	2.31 ± 0.01	0.38	0.08
metoprolol	−0.00612 ± 0.00036	9.50 ± 0.13	1.57 ± 0.01	−0.53	1.15
mianserin	−0.02148 ± 0.00155	7.25 ± 0.03	3.74 ± 0.01	3.50	0.99
naproxen	0.01050 ± 0.00460	4.47 ± 0.09	3.11 ± 0.00	0.27	−1.70
nicotine	0.00897 ± 0.00167	8.09 ± 0.01	1.31 ± 0.01	0.54	0.40
nortriptyline	−0.01133 ± 0.00210	10.05 ± 0.03	4.29 ± 0.01	1.79	1.04
paroxetine	0.02144 ± 0.00689	9.76 ± 0.08	3.39 ± 0.04	1.19	0.48
phenobarbital	−0.01824 ± 0.00855	7.43 ± 0.06	1.26 ± 0.01	0.98	−0.12
phenylbutazone	0.00083 ± 0.00015	4.58 ± 0.05	3.64 ± 0.01	0.96	−0.52
physostigmine	0.01268 ± 0.00087	8.24 ± 0.04	1.69 ± 0.03	0.81	0.08
pindolol	0.00127 ± 0.00052	9.62 ± 0.07	1.91 ± 0.01	−0.28	0.30
promazine	−0.01399 ± 0.00228	9.39 ± 0.00	4.54 ± 0.00	2.55	1.23
propranolol	0.00202 ± 0.00061	9.46 ± 0.01	3.31 ± 0.01	1.29	0.85
quinidine	0.00340 ± 0.00040	8.82 ± 0.03	3.50 ± 0.00	2.08	0.33
ranitidine	−0.00256 ± 0.00041	8.43 ± 0.01	0.34 ± 0.02	−0.75	−1.23
risperidone	0.00698 ± 0.00377	8.27 ± 0.04	3.09 ± 0.03	2.17	−0.02
ropinirole	−0.01252 ± 0.00126	9.70 ± 0.05	3.24 ± 0.01	0.94	0.25
rivastigmine	−0.00018 ± 0.00009	8.91 ± 0.01	2.18 ± 0.01	0.66	0.88
salbutamol	−0.01030 ± 0.00502	9.60 ± 0.01	0.30 ± 0.04	−1.90	−1.03
salicylic acid	0.02255 ± 0.00676	2.79 ± 0.01	2.31 ± 0.01	−0.98	−1.10
thioperamide	0.01849 ± 0.00363	6.90 ± 0.04	2.39 ± 0.02	2.27	−0.16
thioridazine	0.00911 ± 0.00062	9.25 ± 0.01	5.38 ± 0.03	3.54	0.24
trazodone	−0.01080 ± 0.00292	6.83 ± 0.04	2.98 ± 0.00	2.88	0.30
trifluoperazine	0.20277 ± 0.00151	7.88 ± 0.04	6.14 ± 0.01	5.53	1.44
triprolidine	0.01006 ± 0.00233	9.39 ± 0.06	3.87 ± 0.01	1.88	0.78
venlafaxine	−0.01411 ± 0.00205	9.67 ± 0.02	2.97 ± 0.00	0.70	0.48
verapamil	0.00675 ± 0.00054	8.84 ± 0.06	3.89 ± 0.01	2.44	−0.52
zidovudine	0.00428 ± 0.00056	9.51 ± 0.00	0.09 ± 0.01	0.09	−1.00
zolmitriptan	−0.00882 ± 0.00356	9.60 ± 0.05	1.19 ± 0.01	−1.01	−1.40

^1^ Mean ± standard deviation of three independent experiments; ^2^ mean ± standard deviation of three independent experiments (for 0.15 M ISA water-based titrations) or mean ± standard deviation from Yasuda–Shedlovsky extrapolation (for methanol cosolvent in use); ^3^ mean ± standard deviation calculated based on three independent potentiometric titrations conducted with diverse n-octanol contents; ^4^ calculated based on the experimentally obtained pK_a_ and log P data; ^5^ experimental in vivo measurements from rats, data retrieved from literature; ^6^ two pK_a_ values were determined, i.e., 6.04 ± 0.00 and 8.13 ± 0.00, and the latest was used for regression preparation.

**Table 2:** The suppression of electroosmotic flow in a coated capillary. EOF stability confirmed by a low relative standard deviation (RSD) [%].

Capillary/Coating	50 µm Uncoated	50 µm POPC/PS ^2^
µ_EOF_, × 10^−8^ [m^2^ × s^−1^ × V^−1^]	5.79 ^1^	4.68 ^1^
RSD [%]	5.27	5.18
number of measurements	170	170

^1^ Mean value; ^2^ capillary coated with POPC/PS liposomes (80:20 mol%).

## Data Availability

Data is contained within the article. Further inquiries can be directed to the corresponding author.

## Abbreviations

The following abbreviations are used in this manuscript:

ADME	Absorption, Distribution, Metabolism, and Excretion
AUC	Area Under the Curve
BBB	Blood–Brain Barrier
BOSSVS	Bag-of-SFA-Symbols in Vector Space
CA	California
Caco-2	Human Colorectal Adenocarcinoma Cell Line
CE	Capillary Electrophoresis
CEC	Capillary Electrochromatography
CNS	Central Nervous System
CV	Cross-Validation
DAD	Diode Array Detector
DMSO	Dimethyl Sulfoxide
DTW	Dynamic Time Warping
EOF	Electroosmotic Flow
GPU	Graphics Processing Unit
HEPES	4-(2-hydroxyethyl)-1-piperazine ethane sulfonic acid
HPLC	High Performance Liquid Chromatography
ISA	Ionic Strength Adjusted
k-NN	k-Nearest Neighbors
LC-MS	Liquid Chromatography–Mass Spectrometry
LEKC	Liposome Electrokinetic Chromatography
LUVs	Large Unilamellar Vesicles
MDCK-MDR1	Madin–Darby Canine Kidney Cells Expressing MDR1
MEKC	Micellar Electrokinetic Chromatography
ML	Machine Learning
NaN	Not a Number
OLS	Ordinary Least Squares
OOF	Out-Of-Fold
PAMPA-BBB	Parallel Artificial Membrane Permeability Assay for the Blood–Brain Barrier
POPC	1-palmitoyl-2-oleoyl-sn-glycero-3-phosphocholine
PS	Phosphatidylserine
ROC	Receiver Operating Characteristic
Ro5	Rule of Five
RoCNS	Rule for Central Nervous System Drugs
RSD	Relative Standard Deviation
SFA	Symbolic Fourier Approximation
SVL	Supported Vesicular Layer
UV	Ultraviolet
